# The Improvement Effect of Different Doses of Gamma Globulin on the Disease Condition of Infants with Hemolytic Disease of Newborn and Their Effects on Immune Factors in Serum

**Published:** 2020-05

**Authors:** Shuwen HUANG, Lin LIU, Guanglei QIAN, Wenxue LIU, Jialiang WANG, Ming LI, Guang YANG

**Affiliations:** 1. Department of Pediatrics, The People’s Hospital of Zhangqiu Area, Jinan 250200, China; 2. Department of Pharmacy, Qingdao Women and Children’s Hospital, Qingdao 266034, China; 3. Department of Radiology, The People’s Hospital of Zhangqiu Area, Jinan 250200, China; 4. Department of Pharmacy, The Affiliated Hospital of Shandong University of TCM, Shandong Provincial Hospital of Traditional Chinese Medicine, Jinan 250011, China; 5. Department of Pediatrics, Yantaishan Hospital, Yantai 264000, China

**Keywords:** Hemolytic disease of newborn, Gamma globulin, Immune function

## Abstract

**Background:**

To explore the effect of different doses of Gamma Globulin (GG) on the condition of children with Hemolytic Disease of Newborn (HDN) and the influence of immune factors in serum.

**Methods:**

Overall, 180 infants with hemolytic disease of newborn in the People’s Hospital of Zhangqiu Area, Jinan, China from April 2016 to August 2018 were divided into group A (88 cases) and group B (92 cases). Group A was given intravenous low-dose GG on the basis of phototherapy, and group B was given intravenous high-dose GG on the basis of phototherapy. The level of serum total bilirubin of the infants, the levels of CD3+, CD4+, CD8+, IgA, IgG and IgM of the infants, the time of jaundice disappearance and the length of hospital stay, hemoglobin and reticulocyte levels were recorded before treatment and after treatment. The number and condition of adverse reactions were recorded.

**Results:**

After treatment, the levels of TBiL, hemoglobin and reticulocyte, the time of jaundice disappearance and hospital stay in group B were significantly lower than those in group A. The level of immune cells in group B was significantly higher than that in group A after 7 days of treatment, and the levels of IgA / IgG / IgM in group B were significantly higher than those in group A after 28 days of treatment.

**Conclusion:**

Intravenous high-dose GG has a better effect on the condition of neonatal hemolytic disease patients, and more effectively improve the immune function of children.

## Introduction

Hemolytic disease of newborn (HDN) is one of the important reasons that make newborns get diseases and die (1, 2). The incompatibility between Rh blood type of the mother and Rh blood type of the fetus and the incompatibility of ABO blood type are the main reason that leads to HDN, they can injure red blood cells of fetuses, as a result, jaundice, anemia and edema can be seen from newborns and result in death. ABO blood type is very common in newborns, accounting for 85.3 % (3, 4). With the development of medical science, a great progress has been made in the treatment of HDN.

Traditional treatment methods, such as enhanced phototherapy and exchange transfusion of hyperbilirubinemia and red blood cell transfusion of anemia, have been studied and applied, but their effects still need to be improved (5, 6). Gamma Globulin (GG), which is clinically used in drug therapy, can reduce the peak of total bilirubin (TBiL) of full-term neonates and shorten their disease course as well as reduce their need for exchanging transfusion when they are intravenously injected and are treated by phototherapy (7). When gamma globulin (GG) enters into the body, it can combine with Fc receptors and block Fc receptors in the reticuloendothelial system, then the hemolytic process is blocked and the injury of red blood cells is reduced, as a result, bilirubin is reduced (8). There are many studies on the clinical application of GG in the treatment of HDF (9, 10), and the effect is satisfactory. The application of GG as a drug for the treatment of HDN is also increasingly recognized clinically, but research regarding the exact dose and the immune factors in serum is rare.

Therefore, the improvement effect of differencet doses of GG on the disease condition of infants with hemolytic disease of newborn and their effects on immune factors in serum were retrospectively analyzed in this study, in order to provide ideas for the clinical treatment of HDN.

## Materials and Methods

### Study subjects

The data of 180 HDN infants with ABO blood type were retrospectively analyzed, they were diagnosed in the People’s Hospital of Zhangqiu Area Jinan, China. According to the dose, the infants were divided into two groups. Eighty-eight infants were intravenously injected with low-dose GG and treated by phototherapy (group A). Ninety-two infants were intravenously injected with high-dose GG and treated by phototherapy (group B). Inclusion criteria: All infants were diagnosed by pathology and did not have nervous system diseases. The result was proved to be positive by anti-human globulin test and antibody elution test. The serum free antibody was positive. The clinical data of infants were complete.

Exclusion criteria: infants with congenital diseases, hypoxia, hereditary metabolic diseases and complicated with infections were excluded.

This study was approved by the Medical Ethics Committee of our hospital. Family members of all infants were informed and signed an informed consent form.

### Treatment methods

All infants stopped breastfeeding and were fed artificially after they were hospitalized. The infants in group A were intravenously injected with lose-dose GG and treated by phototherapy. Blue lights with a wavelength of 425 nm–475 nm were used in traditional phototherapy group (Beijing Julongsanyou Technology Co., Ltd.) (Phototherapy infant incubators and B-1000G blue lights, China). The phototherapy was carried out, with a light intensity of 10 μW–12 μW / (cm^2^ / nm). The infants were exposed continuously for 12 hours, then they relaxed for 12 hours, next they were exposed for 12 hours again. This step was carried out repeatedly for 3 days. After the above step was finished, the infants were intravenously injected with GG once a day, the concentration of GG was 400 mg/kg (Manufacturer: Shanxi Kangbao Biological Product Co., Ltd.; SFDA approval number S19994004). This step was carried out repeatedly for 3 days. The infants in group B were intravenously injected with high-dose GG and treated by phototherapy. The phototherapy was carried out according to the steps that were used in group A. The infants were intravenously injected with GG once a day, the concentration of GG was 1000 mg/kg. This step was carried out repeatedly for 3 days.

### Observation indicators

The general data of the infants were recorded, including gender, age, weight, gestational age, ABO blood type, etc. The differences in those data were compared. The levels of TBiL, hemoglobin (Hb) and reticulocytes (RetB) of the infants were recorded before treatment and in 24 h, 48 h, and 72 h after treatment. The levels of CD3+, CD4+, CD8+, IgA, IgG and IgM of the infants were recorded before treatment and in 7 days and 28 days after treatment. The appearance time and disappearance time of the infants’ jaundice and their hospitalization time were recorded. The number of the infants who had adverse reactions and their condition were recorded.

### Statistical methods

SPSS19.0 was used (Asia Analytics Formerly SPSS China). The measurement data were expressed in the form of %. The ratio between two groups was compared by χ^2^ test. The count data were expressed in the form of mean ± standard deviation (mean ± sd). The comparison between two groups was performed by independent sample t-test. *P*<0.05 meant the difference was statistically significant.

## Results

### General data

The age of the infants in two groups was between 0 day old and 2 days old, and their weight was between 2.8 kg and 4.5 kg when they were born. There were 88 infants in group A, in which there were 43 males (48.86%) and 45 females (51.14%). The age of the infants was (1.01±0.24) days old. There were 48 males (52.17%) and 44 females (47.83%) in group B. The age of the infants was (0.98±0.31) days old. The differences in gender, age, weight, gestational age and other data of the infants in two groups were not statistically significant (Table 1).

**Table 1:** General data of participants

***Variable***	***Group A(n=88)***	***Group B (n=92)***	***χ^2^/t***	***P***
Gender [n(%)]			0.197	0.657
Male	43(48.86)	48(52.17)		
Female	45(51.14)	44(47.83)		
Age(d)	1.01±0.24	0.98±0.31	0.724	0.470
Weight (kg)	3.21±0.56	3.12±0.42	1.223	0.223
Way of delivery			0.283	0.590
Eutocia	56 (63.64)	55 (59.78)		
Cesarean delivery	32 (36.36)	37 (40.22)		
Gestational age(week)	35.14±1.56	35.56±1.43	1.884	0.061
Blood type [n(%)]				
A	23(26.14)	22(23.91)	0.119	0.731
B	18(20.45)	23(25.00)	0.528	0.467
AB	22(25.00)	25(27.18)	0.110	0.740
O	25(28.41)	22(23.91)	0.471	0.492
Serum cystatin C(mg/L)	1.35±0.27	1.42±0.21	1.946	0.053
Urine cystatin C(mg/L)	0.68±0.49	0.72±0.42	0.589	0.557

### The analysis of the level of serum total bilirubin

The difference in the level of TBiL of the infants between group A and group B was not statistically significant before they were treated (*P*=0.600). The level of TBiL of the infants in the two groups in 24 hours, 48 hours, and 72 hours after they were treated was lower than that of the infants within the group before they were treated (*P*<0.001), and the level of TBiL gradually decreased over time. After treatment, the level of TBiL in group B was significantly lower than that in group A (*P<*0.05*)* (Table 2).

**Table 2:** The analysis of the level of serum total bilirubin (μmol/L)

***Variable***	***Group A (n=88)***	***Group B (n=92)***	**t**	**P**
Before treated	312.21±40.64	315.30±38.21	0.526	0.600
In 24 hours after treated	266.24±35.23^*^	242.66±38.24^*^	4.297	<0.001
In 48 hours after treated	224.21±34.36^*#^	178.56±36.65^*#^	8.612	0.492
In 72 hours after treated	183.35±32.45^*#@^	125.88±35.26^*#@^	11.364	0.618
F	214.7	175.24		
*P*	<0.001	<0.001		

Note:

* means compared to the infants in the same group before they were treated, *P*<0.05.

# means compared to the infants in the same group in 24 hours after they were treated, *P*<0.05.

@ means compared to the infants in the same group in 48 hours after they were treated, *P*<0.05

### The analysis of the levels of CD3+, CD4+, and CD8+

There was no significant difference in the levels of CD3+, CD4+, and CD8+ of the infants in two groups before they were treated. The levels of CD3+, CD4+, and CD8+ of the infants in group B were significantly higher than those in group A (*P*=0.004, *P*=0.020, *P*=0.013). There was no significant difference in the levels of CD3+, CD4+, and CD8+ of the infants in two groups in 28 days after they were treated (*P*=0.901, *P*=0.759, *P*=0.917). The levels of CD3+, CD4+ and CD8+ in the two groups at 7 days after treatment and 28 days after treatment were significantly higher than those before treatment (*P*<0.05). The levels of CD3+, CD4+ and CD8+ at 28 days after treatment were significantly higher than those at 7 days after treatment (*P*<0.05) (Table 3).

**Table 3:** The analysis of the level of CD3+, CD4+ and CD8+ (number / μL)

***Variable***	***Group A (n=88)***	***Group B (n=92)***	**t**	**P**
CD3+				
Before treated	852.25±335.01	865.87±344.12	0.269	0.789
In 7 days after treated	906.54±338.98^*^	1055.72±354.32^*^	2.884	0.004
In 28 days after treated	1128.74±356.28^*#^	1135.29±351.65^*#^	0.124	0.901
F	16.24	13.76		
*P*	<0.001	<0.001		
CD4+				
Before treated	652.25±225.88	648.47±220.69	0.114	0.910
In 7 days after treated	712.28±230.55^*^	793.45±236.78^*^	2.329	0.02
In 28 days after treated	1025.69±266.35^*#^	1037.45±268.47^*#^	0.295	0.768
F	60.63	60.31		
*P*	<0.001	<0.001		
CD8+				
Before treated	554.74±220.65	546.95±219.45	0.237	0.813
In 7 days after treated	646.23±221.85^*^	728.87±217.61^*^	2.523	0.013
In 28 days after treated	758.69±235.32^*#^	762.36±233.02^*#^	0.105	0.917
F	17.97	24.75		
*P*	<0.001	<0.001		

Note:

* means compared to the infants in the same group before they were treated, *P*<0.05;

# means compared to the infants in the same group in 7 days after treated, *P*<0.05

### The analysis of the levels of IgA, IgG and IgM

There was no significant difference in IgG levels between the two groups before treatment. After 7 days of treatment and 28 days after treatment, the levels of IgA, IgG and IgM in group B were significantly higher than those in group A (*P*<0.05). The levels of IgA, IgG and IgM in the two groups were significantly higher at 28 days after treatment than those at 7 days after treatment (*P*<0.05). The IgG levels in the two groups at 7 days after treatment were significantly higher than those before treatment (*P*<0.05) (Table 4).

**Table 4:** The analysis of the level of IgA, IgG and IgM (number / μL)

***Variable***	***Group A (n=88)***	***Group B (n=92)***	**t**	**P**
IgA				
Before treated	—	—	—	—
In 7 days after treated	1.52±0.58	2.24±0.84	6.663	<0.001
In 28 days after treated	2.74±1.27^#^	3.89±1.78^#^	4.970	<0.001
t	8.19	8.04		
*P*	<0.001	<0.001		
IgG				
Before treated	4.62±1.88	4.49±1.97	0.453	0.651
In 7 days after treated	6.17±2.41^*^	8.32±2.67^*^	5.663	<0.001
In 28 days after treated	9.15±3.27^*#^	11.68±3.59^*#^	4.936	<0.001
F	19.84	14.95		
*P*	<0.001	<0.001		
IgM				
Before treated	—	—	—	—
In 7 days after treated	1.10±0.34	1.79±0.58	9.681	<0.001
In 28 days after treated	2.56±1.25^#^	3.54±1.92^#^	4.039	<0.001
*t*	10.57	8.19		
*P*	<0.001	<0.001		

Note:

* means compared to the infants in the same group before they were treated, *P*<0.05;

# means compared to the infants in the same group in 7 days after treated, *P*<0.05

### The analysis of the appearance time and disappearance time of the infants’ jaundice and their hospitalization time

**Fig. 1:**
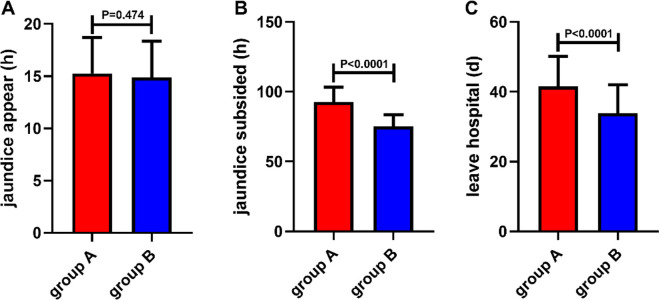
Analysis of the appearance and disappearance time of jaundice and hospitalization time. The disappearance time of jaundice and hospitalization time in group B were significantly lower than those in group A. *, *P*>0.05

The occurrence time of jaundice in group A and group B was (15.25±3.45) h and (14.88±3.47) h, respectively, and the disappearance time of jaundice in group A and group B was (92.52±10.62) h and (75.34±8.21) h, respectively. The hospitalization time was (41.55±8.56) d and (33.82 ± 8.15) d, respectively. There was no significant difference in the appearance and disappearance time of jaundice between the two groups). The disappearance time of jaundice and hospitalization time in group B were significantly lower than those in group A (*P*<0.05) (Fig. 1).

### The analysis of the levels of Hb and RetB

The differences in the levels of Hb and RetB of the infants in group A and group B were not statistically significant before they were treated. The levels of Hb and RetB of the infants in group A and group B in 24 hours, 48 hours, and 72 hours after they were treated were lower than those of the infants in two groups before they were treated, and the levels of Hb and RetB of them gradually decreased over time.

After treatment, the levels of Hb and RetB in group B were significantly lower than those in group A, and the difference was statistically significant (*P*<0.05) (Table 5).

**Table 5:** The analysis of the levels of Hb and RetB

***Variable***	***Group A (n=88)***	***Group B (n=92)***	**t**	**P**
HHb (g/L)				
Before treated	172.54±12.65	173.14±13.21	0.311	0.756
In 24 hours after treated	162.42±11.63^*^	154.69±12.09^*^	0.413	0.680
In 48 hours after treated	150.36±12.44^*#^	135.84±13.54^*#^	1.793	0.075
In 72 hours after treated	130.68±10.33^*#@^	113.65±10.25^*#@^	0.639	0.524
F	20.48	39.29		
*P*	<0.001	<0.001		
RetB (%)				
Before treated	11.32±1.56	11.13±1.77	0.763	0.447
In 24 hours after treated	10.45±1.28^*^	8.57±1.63^*^	0.548	0.585
In 48 hours after treated	8.82±1.42^*#^	7.18±1.39^*#^	0.764	0.446
In 72 hours after treated	7.45±1.63^*#@^	5.42±1.46^*#@^	0.608	0.544
F	11.91	21.70		
*P*	<0.001	<0.001		

Note:

* means compared to the infants in the same group before they were treated, *P*<0.05.

# means compared to the infants in the same group in 24 hours after they were treated, *P*<0.05.

@ means compared to the infants in the same group in 48 hours after they were treated, *P*<0.05

### The analysis of adverse reactions

**Fig. 2:**
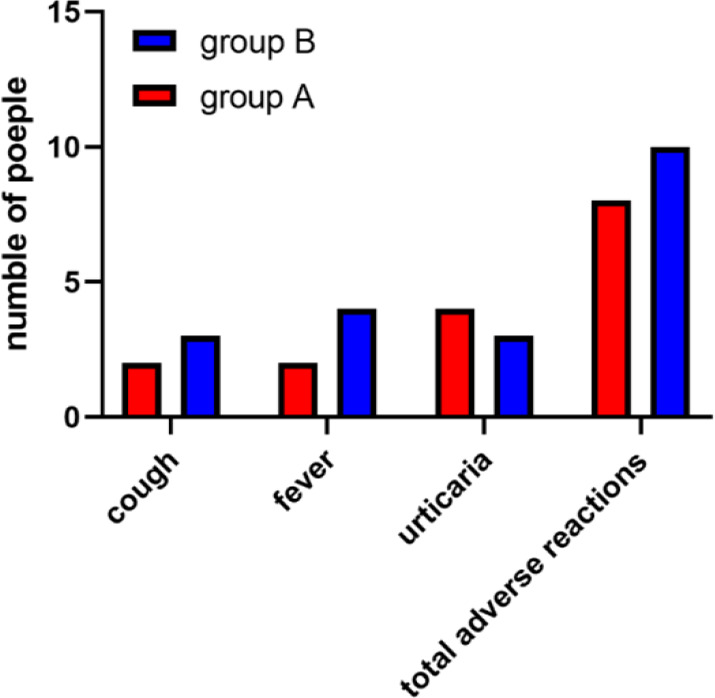
Analysis of adverse reactions. There were no significant differences in the adverse reactions of cough, fever, and urticaria between the two groups

In group A, there were 8 cases (9.09%) with adverse reactions, including 2 cases of cough (2.27%), 2 cases of fever (2.27%), 4 cases of urticaria (4.54%). While in group B, there were 10 cases (10.87%) with adverse reactions, including 3 cases of cough (3.26%), 4 cases of fever (4.35%), 3 cases of urticaria (3.26%). There were no significant differences in the adverse reactions of cough, fever and urticaria between the two groups. There was no significant difference in the total adverse reactions. No serious adverse reactions occurred in the two groups (Fig. 2).

## Discussion

The incompatibility of ABO blood type easily leads to the occurrence of HDN (11, 12). HDN occurs when red blood cells or blood type antibodies of the mother enter into the fetus through the placenta and then injure red blood cells of the fetus during the gestation period. HDN likely leads to edema, ascites, heart failure, and death. For the fetus whose disease condition is not serious, the incompatibility of red blood cells still exists after the fetus is born. It is reported that the morbidity of HDN is between 0.33% and 2.2%. As the morbidity of HDN is high, it needs to be treated effectively in clinic (13). In addition to phototherapy, GG has been recognized in clinic, there are many reports about the efficacy of them. Some studies indicate that GG can reduce patients’ need for exchanging transfusion and reduce the complications related to exchange transfusion (14). Hemolytic disease of a 35-year-old and Rh-negative pregnant woman with Rh allogeneic immunity was treated successfully by plasmapheresis, intravenous injection of GG, and intrauterine transfusion (15). Studies about the exact dose and immune factors in serum are rare. Therefore, the improvement effect of different doses of GG on the disease condition of infants with hemolytic disease of newborn and their effects on immune factors in serum were retrospectively analyzed in this study, in order to provide ideas for the clinical treatment of HDN.

The levels of TBiL in group A and group B after 24, 48, and 72 hours of treatment were lower than those in the same group before treatment, and gradually decreased with time. After treatment, the level of TBiL in group B was significantly lower than that in group A. After 7 days of treatment, the levels of CD3+, CD4+, and CD8+ in group B were significantly higher than those in group A. There was no significant difference in CD3+, CD4+, and CD8+ levels between the two groups after 28 days of treatment. The levels of CD3+, CD4+, and CD8+ in the two groups at 7 days and 28 days after treatment were significantly higher than those before treatment. The levels of CD3+, CD4+, and CD8+ were significantly higher at 28 days after treatment than those at 7 days after treatment; after 7 days of treatment and 28 days after treatment, the levels of IgA, IgG and IgM in group B were significantly higher than those in the group A. The levels of IgA, IgG and IgM in the two groups at 28 days after treatment were significantly higher than those in the 7 days after treatment. The IgG levels in the two groups in the 7 days after treatment were significantly higher than those before treatment. The results indicated that high-dose GG could effectively increase the levels of CD3+, CD4+, and CD8+ in children in a short period of time compared with low doses. After a certain period of time, the levels of CD3+, CD4+, and CD8+ in the two groups would maintain a certain level. In referring to the IgA, IgG, and IgM levels, high-dose GG was significantly better than the low-dose group. In addition, the disappearance time of jaundice and hospitalization time in group B were significantly lower than those in group A. The levels of Hb and RetB in group B were significantly lower than those in group A after treatment. There was no significant difference in adverse reactions during treatment. This indicates that high dose GG is better at improving HDF efficacy and immune factor levels.

High doses of GG could control the disease more effectively and reduce blood exchange, but the effect of GG on immune factors in the body was not studied (16). The fetal immune system is still immature and relies mainly on the components of the innate immune system or antigen-independent immune system (17). In case of hemolysis, intravenous GG can has a dual role in immunosuppression and immune enhancement, by blocking reticuloendothelial system FC receptors and anti-SARA antibody (Glycoprotein produced by proliferation and differentiation of B cells into plasma cells after antigen stimulation), to prevent it from binding to SARA erythrocyte antigens, thereby preventing red blood cell damage from acting (18, 19). At the same time, the body’s immune was improved.

When mothers whose fetus has hemolytic disease are intravenously injected with GG, the clinical effect of GG on the disease course and severity of hemolytic disease of the fetus is potentially good, and that the appearance time of anemia of the fetus in pregnant women who are intravenously injected with GG is later than that of anemia of the fetus in pregnant women who are not intravenously injected with GG. In the above study, it is believed that patients with the history of serious diseases should be treated with GG as early as possible (<13 weeks) (20, 21). Unfortunately, when GG is used to treat hemolytic disease of infants, the morbidity of necrotizing colitis will increase, but the mortality will not increase. When GG is used to treat hemolytic disease, the morbidity of intestinal circulation injury will increase and the morbidity of appendicitis will increase (22). Two patients with GG treated hemolysis had abnormal heart rhythm, and it is recommended that the cardiopulmonary function of patients should be examined when they are treated with GG (23). In our study, we did not find a similar situation, which we believe is independent of the dose, but still needs to be paid attention to clinically.

## Conclusion

The disease condition of infants with hemolytic disease of newborn will be improved when they are intravenously injected with high-dose GG, and GG improves their immune function.

## Ethical considerations

Ethical issues (Including plagiarism, informed consent, misconduct, data fabrication and/or falsification, double publication and/or submission, redundancy, etc.) have been completely observed by the authors.
